# Molecular Recognition between Carbon Dioxide and Biodegradable Hydrogel Models: A Density Functional Theory (DFT) Investigation

**DOI:** 10.3390/gels10060386

**Published:** 2024-06-05

**Authors:** Domingo Cesar Carrascal-Hernandez, Maximiliano Mendez-Lopez, Daniel Insuasty, Samira García-Freites, Marco Sanjuan, Edgar Márquez

**Affiliations:** 1 Departamento de Química y Biología, Facultad de Ciencias Básicas, Universidad del Norte, Barranquilla 080020, Colombia; domingoh@uninorte.com (D.C.C.-H.); maximilianom@uninorte.edu.co (M.M.-L.); insuastyd@uninorte.edu.co (D.I.); 2 Centro de Investigación e Innovación en Energía y Gas—CIIEG, Promigas S.A. E.S.P., Barranquilla 11001, Colombia; samira.garcia@promigas.com (S.G.-F.); marco.sanjuan@promigas.com (M.S.)

**Keywords:** carbon dioxide, CO_2_-capture, frontier molecular orbitals, green-hydrogen, DFT

## Abstract

In this research, we explore the potential of employing density functional theory (DFT) for the design of biodegradable hydrogels aimed at capturing carbon dioxide (CO_2_) and mitigating greenhouse gas emissions. We employed biodegradable hydrogel models, including polyethylene glycol, polyvinylpyrrolidone, chitosan, and poly-2-hydroxymethacrylate. The complexation process between the hydrogel and CO_2_ was thoroughly investigated at the ωB97X-D/6-311G(2d,p) theoretical level. Our findings reveal a strong affinity between the hydrogel models and CO_2_, with binding energies ranging from −4.5 to −6.5 kcal/mol, indicative of physisorption processes. The absorption order observed was as follows: chitosan > PVP > HEAC > PEG. Additionally, thermodynamic parameters substantiated this sequence and even suggested that these complexes remain stable up to 160 °C. Consequently, these polymers present a promising avenue for crafting novel materials for CO_2_ capture applications. Nonetheless, further research is warranted to optimize the design of these materials and assess their performance across various environmental conditions.

## 1. Introduction

With the rapid development of industrialization and urbanization, there has been a significant increase in the emission of greenhouse gases into the atmosphere [1]. This increase in emissions is largely due to human activities such as burning fossil fuels, deforestation, and changing agricultural and industrial practices. As a result, the buildup of greenhouse gases has caused global warming and climate change, which have had severe consequences such as rising sea levels, extreme weather events, and loss of biodiversity. The impact of climate change is not limited to the environment but also affects human welfare, causing public health issues and economic losses. It is essential to take coordinated action at the global, regional, national, and local levels to mitigate the impact of climate change and make cities an integral part of the solution [2].

To address the challenge of climate change, the technology known as carbon capture, utilization, and storage (CCUS) has been proposed. This technique allows for the capture of CO_2_ from emission sources, including power plants, industrial facilities, and the atmosphere. Once captured, CO_2_ can be utilized as a raw material for chemical synthesis or stored deep underground in a safe and permanent manner [3]. This innovative approach offers the potential to achieve net-zero emissions on a large scale. CCUS, which includes biomass power with carbon capture and storage (BECCUS) when biomass is used, can be implemented in existing coal and gas power plants. By capturing CO_2_ from these emission sources, as well as directly from the atmosphere, it becomes possible to utilize the captured CO_2_ for chemical synthesis or store it securely underground for long-term storage [4]. In addition to contributing to the electricity supply sector, CCUS is possibly the only scalable and cost-effective option to achieve a deep decarbonization of certain industries such as steel, cement, glass, and ceramics, as well as the manufacture of chemical products that generate CO_2_ during production processes [5]. The analyses carried out by the Intergovernmental Panel on Climate Change (IPCC) and the International Energy Agency (IEA) have shown that CCUS will be key to achieving “Net Zero” in 2050, which contributes to a sixth of the reduction of global CO_2_ emissions in order to limit the increase in global temperature to less than 1.5 °C, as established in the Paris Agreement [6].

Carbon capture technologies have been developed in recent decades, and can be divided into three pathways: post-combustion capture, oxy-combustion, and pre-combustion capture [7]. Various physical and chemical processes have been reported to be used in carbon capture technology, such as solvent-based adsorption, solid solvent adsorption/absorption, membranes, cryogenics, and chemical loops for CO_2_ separation [8,9,10]. Among these methods, chemical absorption is currently one of the most widely used and commercially available techniques [11,12]. However, there are several limitations, such as the high energy consumption required for solvent regeneration, as well as the corrosiveness, high toxicity, volatility and high cost of solvents, which are the main barriers to the deployment of carbon capture technology carbon [13]. 

In this sense, the use of biodegradable hydrogels for CO_2_ absorption is an interesting approach that has attracted attention in recent research. The advantages of using biodegradable hydrogels in CO_2_ absorption include their biodegradability, which makes them more environmentally friendly than traditional materials [14]. Furthermore, hydrogels can be synthesized to exhibit CO_2_ responsive properties, and swell or deflate in the presence of CO_2_, further enhancing their potential for CO_2_ capture applications [13]; however, the selection of the most suitable absorbent hydrogels remains a challenge. 

In light of the aforementioned challenges, our study introduces a novel approach to CO_2_ absorption using Density Functional Theory (DFT) to predict the most effective absorbent hydrogels. This innovative method stands out as it allows for a more precise and efficient selection of hydrogels, thereby overcoming the existing challenge in the field [15,16]. By leveraging DFT, we are able to model and predict the interaction between CO_2_ and potential absorbent materials at the atomic and molecular levels. This provides a more detailed understanding of the absorption process, enabling the design of more efficient and effective hydrogels. Furthermore, our approach also considers the environmental impact and cost-effectiveness of the absorbent materials, ensuring a sustainable and economically viable solution to CO_2_ capture [17,18]. This work, therefore, not only contributes to the advancement of CCUS technology but also paves the way for the development of next-generation absorbents for greenhouse gas capture.

Molecular recognition is a pivotal factor influencing the selectivity of CO_2_-absorbent materials. The paramount significance of frontier molecular orbitals (FMOs) in molecular recognition cannot be overstated. These FMOs precisely delineate the regions where chemical bonds exhibit heightened reactivity owing to their associated orbital energies. Furthermore, molecular orbital theory appropriately elucidates the selectivity of chemical reactions and the formation of discrete product species [19,20].

Given the significant role of molecular frontier orbitals in molecular recognition, their utility in elucidating the selectivity of CO_2_-absorbent materials is substantial. This understanding serves as the foundation for our research, which endeavors to engineer biodegradable hydrogels for CO_2_ absorption using Density Functional Theory (DFT).

Our study employs concepts such as binding energy, frontier molecular orbitals, and chemical bonds, and utilizes poly(2-hydroxyethylmethacrylate), poly(ethylene glycol), chitosan, and polyvinylpyrrolidone as models of CO_2_ bio-sorbent hydrogels. These hydrogels have demonstrated a commendable capacity for CO_2_ absorption [21,22,23]. 

This methodological approach will facilitate the validation of DFT as a predictive tool and stimulate the synthesis of hydrogel matrices with enhanced CO_2_ absorption capacity. 

DFT, a widely accepted method for understanding and predicting the adsorption of a specific molecule on a polymeric structure, has been shown to yield results that correlate satisfactorily with experimental outcomes [24,25]

Therefore, the study of the relative absorption capacity of biodegradable hydrogels for CO_2_ absorption was carried out using the concepts of interaction energy and Gibbs energy. The nature of molecular interactions was studied using frontier molecular theory, binding energy, and the Gibbs energy equation. This work underscores the critical role of frontier molecular orbitals in designing effective CO_2_-absorbent materials, thereby emphasizing the importance of this study.

## 2. Results and Discussion 

### 2.1. Minimum-Energy Structures

**Figure 1:**
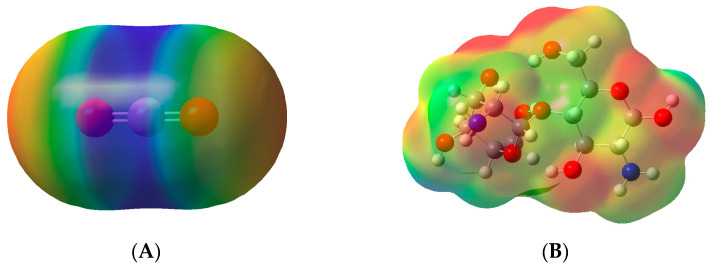

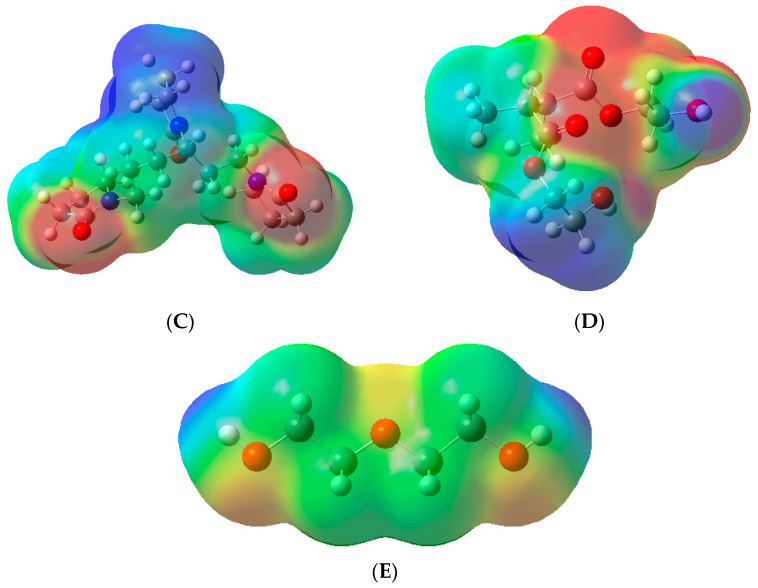
Electrostatic surface potential (ESP). (**A**): carbon dioxide; (**B**): chitosan; (**C**): polyvinylpyrrolidone; (**D**): 2-hydroxyethyl acrylamide and (**E**): polyethyleneglycol.

The minimum-energy structures for the dimer were selected from the results of the systematic rotor search; the global minimum-energy structures were optimized at ωB97X-D/6-311G(2d,p) and verified using frequency calculations. The global minimum structures for dimer along with CO_2_ are shown in Figure 1 embedded in their electrostatic potential (ESP) surface. The electrostatic surface potential is crucial for CO_2_ molecular recognition by hydrogels. It enables the design of hydrogel materials that can selectively and efficiently capture CO_2_, offering promising solutions for carbon capture and reduction of greenhouse gas emissions [26,27]. 

As shown by the results, except for carbon dioxide, all the compounds studied herein are polar in nature. Even though CO_2_ are non-polar, the molecule is polarizable due to the presence of O in the extreme of the molecule; therefore, in the presence of polar compounds, CO_2_ could polarize its electron-cloud and participate in dipole-induced dipole interaction or hydrogen bond acceptor [28,29]. 

Chitosan, polyvinylpyrrolidone, and 2-hydroxyethylacrylamide showed electron density distributions characterized by pronounced asymmetry. This property significantly enhances their propensity for dipole–dipole interactions. Conversely, polyethene glycol exhibits a more symmetrical electron density distribution, marked by the presence of hydrogen atoms bearing partial positive charges (depicted in blue). These hydrogen atoms are pivotal for hydrogen bonding interactions. Collectively, these findings suggest a heightened likelihood of induced dipole–dipole interactions with carbon dioxide (CO_2_), thereby promoting its absorption.

### 2.2. Minimum-Energy Structures for Dimer and CO_2_-Hydrogel Complexes: Molecular Recognition

Molecular recognition is a fundamental concept in the chemical industry that involves specific interactions between molecules based on complementary shapes, sizes, charges, and functional groups [30,31]. It plays a crucial role in various aspects of the chemical industry, primarily in the field of drug discovery, material and engineering design, catalysis, separation technology, sensor development, and supramolecular chemistry, among others [31,32]. The relationship between frontier molecular orbitals and molecular recognition lies in how the electronic structure of molecules, described by these orbitals, influences their ability to interact and recognize each other specifically [33,34]. Frontier molecular orbitals, consisting of the highest occupied molecular orbital (HOMO) and the lowest unoccupied molecular orbital (LUMO), play a crucial role in molecular recognition [35,36]. 

The energy gap between the HOMO and LUMO orbitals determines the ease of electron transfer between molecules. In molecular recognition, this energy gap influences the strength of the interactions between molecules. When two molecules approach each other, their HOMO and LUMO orbitals can overlap, allowing charge transfer interactions. A smaller HOMO–LUMO gap improves electron transfer, affecting the strength of recognition interactions [36].

In this context, the investigation of frontier orbitals at the interface between dimers and CO_2_ emerges as a critical analytical approach. This exploration provides invaluable insights into the feasibility of CO_2_ absorption within a given polymeric matrix, constituting a fundamental step in the selection of the optimal absorbent material. 

**Figure 2:**
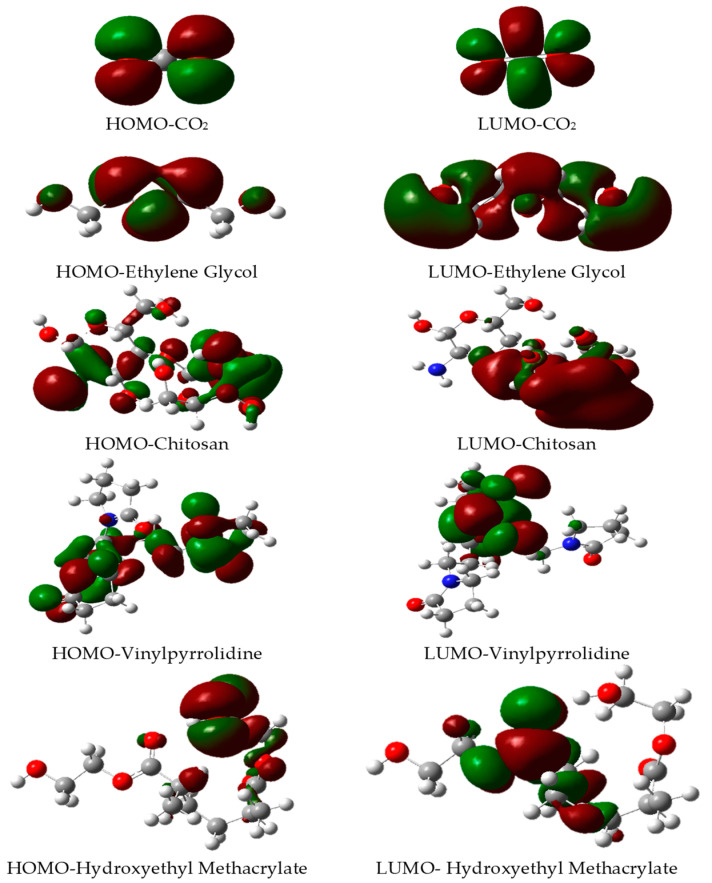
Frontier molecular orbitals (HOMO and LUMO) for all compounds studied herein, obtained at ωB97X-D/6-311G(2d,p).

Figure 2 illustrates the frontier orbitals of the candidate absorptive compounds, including vinylpyrrolidone, ethylene glycol, hydroxyethyl methacrylate, and chitosan, alongside the target absorbate, CO_2_. Notably, the frontier orbitals of CO_2_ exhibit a remarkable degree of uniformity, with electronic density uniformly distributed throughout its molecular structure. This characteristic renders CO_2_ highly versatile, and capable of functioning as both an electron acceptor and donor—a trait that augments its propensity for interaction with a wide array of chosen biodegradable matrices.

Notably, the frontier orbitals of chitosan, polyvinylpyrrolidone, and ethylene glycol share striking similarities with those of CO_2_. This observation underscores the potential for efficient absorption of CO_2_ gas onto the polymeric matrices formed by these compounds. Such molecular-level compatibility hints at the possibility of establishing strong intermolecular interactions between the polymeric matrices and CO_2_ molecules, suggesting these materials as promising candidates for CO_2_ capture and storage applications. This intricate understanding of frontier orbital interactions not only facilitates the selection of the most suitable absorbent but also contributes significantly to the advancement of environmentally sustainable solutions for managing greenhouse gas emissions.

### 2.3. Hydrogels Model CO_2_ Complexation

**Figure 3:**
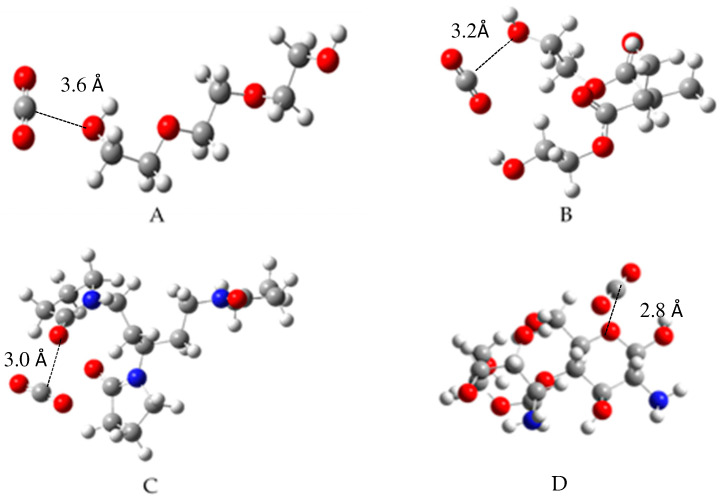
Minimum molecular structure for complexes of: (**A**): CO_2_–polyethyleneglycol; (**B**): CO_2_–2-hydroximethacrylate; (**C**): CO_2_–polyvinylpyrrolidone; (**D**): CO_2_–chitosan.

Once it was established that symmetry existed between the frontier orbitals, the search for the minimum energy configuration in the formation of the complex between CO_2_ (the absorbate) and the hydrogel model (the absorbent) was studied. To attain this minimum energy configuration, a systematic rotor search was conducted in tandem with a genetic algorithm to identify the global minimum. Subsequently, these complexes were saved in .gjf file format and optimized using Gaussian 16. Their optimization was verified through a frequency calculation at the ωB97X-D/6-311G(2d,p) theory level. The structures with the lowest energy levels are depicted in Figure 3. In all instances, the CO_2_ molecule is oriented such that the carbon atom is proximate to an electronegative atom, such as oxygen or nitrogen, at interatomic distances of 2.8–3.6 Å. This suggests short-range interactions, such as dipole–dipole or hydrogen bonding; therefore, a Van der Waals interaction seemed imminent. It is noteworthy that in the case of chitosan and PVP, the interatomic distances are smaller, which aligns with the Highest Occupied Molecular Orbitals (HOMO) shape in both cases. This implies a superior molecular interaction with the CO_2_ molecule, facilitating its absorption.

### 2.4. Thermodynamics of Molecular Complexation

Molecular recognition occurs through an orbital interaction, specifically, the Highest Occupied Molecular Orbital (HOMO) of one participant must interact with the Lowest Unoccupied Molecular Orbital (LUMO) of the other species. Once the structures of the frontier orbitals have been examined, suggesting a probable molecular recognition, it becomes necessary to determine the interaction energy. To do this, as explained in the methodology, we used binding energy. A negative value implies a spontaneous attraction between the species, signifying that molecular recognition takes place spontaneously. The feasibility of this interaction and the potential adsorption of the absorbate into the absorbent matrix depend on the magnitude of this parameter. Thus, values between −50 and −1 kJ/mol would indicate physisorption, meaning CO_2_ is absorbed without the presence of a covalent bond [37,38]. These results are qualitatively in line with some experimental works [21,22,23].

Table 1 shows that all ΔEb (binding energy difference) values are negative, indicating an attractive interaction. The enthalpy (H) values suggest that the absorbent–absorbate complex formation is exothermic; the entropy change (S) suggests a diminishing of disorder during the complexes’ formation; chitosan and PVP are the dimers with the major molar volume; in addition are the models with the more negative values of H, suggesting a greater molecular interaction between CO_2_ therefore, As expected, these two complexes (chitosan–CO_2_ and PVP–CO_2_) showed the greater change in entropy values. 

The Gibbs free energy (G) values confirm that the processes are spontaneous. 

In other words, there is a high probability that the hydrogels constructed from these monomers will absorb CO_2_ through physisorption, as corroborated by the magnitude of these values [39]. It is important to note that, based on these results, and knowing that since Gibbs energy is connected to the equilibrium constant through the formula ΔG° = −RTlnKe [40], the order of CO_2_ absorption would be chitosan > PVP > HEAC > PEG. Interestingly, all the first three compounds in the mentioned order contain nitrogen atoms in their molecular structure, demonstrating the significance of this atom in the absorption and transformation of CO_2_. 

The last two columns are related to the absorption predicted for the methods used herein and their experimental reports as CO_2_-absorbent. Interesting, chitosan, PVP, and PEG are reported as CO_2_-absorbent; therefore, the methodology used herein qualitatively reproduce the experimental results, suggesting a good starting point to predict new hydrogel matrices for CO_2_ absorption. On the other hand, in the best of our knowledge, HEAC is not reported as a single hydrogel matrix for CO_2_ absorption; however, the results presented herein suggest it could be a good CO_2_ absorbent.

Computational calculations identify chitosan as the most promising absorbent. Chitosan offers the advantage of having more nitrogen groups in its structure, and possesses a more suitable reticular structure that facilitates the diffusion of CO_2_ through the polymeric matrix [41]. Additionally, it is obtained from natural sources, making its natural degradation much more spontaneous than the other monomers listed in Table 1. However, the use of other monomers is not ruled out, and it could be interesting to explore their potential in CO_2_ capture applications in future.

### 2.5. Temperature Dependence of Complexation Gibbs Energy

Given that CO_2_ is a gas, it is anticipated that its absorption capacity will diminish as the temperature rises [37,42]. This phenomenon can be attributed to the thermodynamics of the exothermic CO_2_ absorption system, which may induce reversible reactions at elevated temperatures. Consequently, it becomes pertinent to calculate the transition temperature, denoted as the temperature at which ΔG (Gibbs free energy change) equals zero.

The Gibbs free energy, which determines the equilibrium conditions of chemical reactions and materials stability, depends on temperature, and changing the temperature can change the sign of ΔG; in this sense, the variation of free energy with temperature for the complexation of CO_2_ absorbance was calculated by the already known Helmholtz equation, and the results are shown in Table 2.

Table 2 shows the values of Gibbs free energy in a temperature range of 25 to 200 °C. The Gibbs free energy decreases as the temperature increases and becomes zero (transition temperature), around 160 °C in each case. This result means that CO_2_ is released above this temperature; that is, after this temperature, the complex becomes unstable. The value of T of transition (the temperature at which the process begins to become non-spontaneous), around 160 °C, indicates that from this temperature, any amount of CO_2_ begins to separate from the polymeric matrix. This temperature seems suitable for the use of these polymeric matrices as absorbents in the post-combustion process of green hydrogen.

## 3. Conclusions

In conclusion, the application of density functional theory (DFT) in designing biodegradable hydrogels for carbon dioxide (CO_2_) capture presents a significant advancement in the field of materials science. The results obtained in this work are qualitatively in line with experimental results. Through the exploration of molecular interactions, frontier molecular theory, and thermodynamic parameters, this study has shed light on the potential of polymeric matrices such as poly(2-hydroxyethyl methacrylate), poly(ethylene glycol), chitosan, and polyvinylpyrrolidone in effectively absorbing CO_2_.

The findings of this research not only suggest the stability of the complexes formed between the hydrogel models and CO_2_ but also suggest promising prospects for the development of novel materials for CO_2_ capture applications. The calculated binding energies and Gibbs energy equations not only are qualitatively in line with experimental results but provide valuable insights into the affinity between the hydrogel models and CO_2_, emphasizing the critical role of frontier molecular orbitals in designing efficient CO_2_-absorbent materials. These methodological approaches offer a theoretical framework for future research and development in the design of eco-friendly materials for mitigating greenhouse gas emissions.

Overall, the outcomes of this study contribute to the growing body of literature on carbon capture technologies and highlight the potential of biodegradable hydrogels as sustainable solutions for addressing climate change. Further optimization and exploration of these materials across various environmental conditions are warranted to maximize their performance and applicability in real-world CO_2_ capture scenarios.

By emphasizing the significance of this research in advancing the development of environmentally friendly materials and its implications for CO_2_ mitigation strategies, this study paves the way for future investigations and innovations in the field of materials science and sustainable technology.

## 4. Materials and Methods

### 4.1. Molecular Modelling for Polymers with High CO_2_ Absorption Capacity

**Figure 4:**
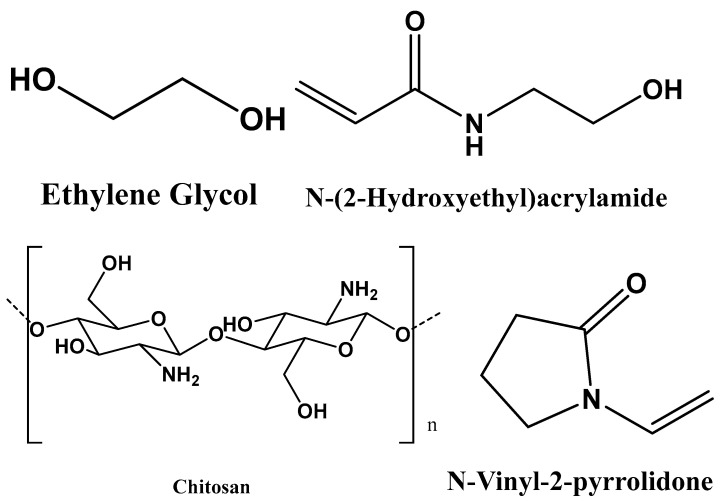
2D molecular structures of monomers.

The study aimed to design biodegradable hydrogels for CO_2_ absorption using Density Functional Theory (DFT). The polymeric matrices, including poly(2-hydroxyethyl methacrylate), poly(ethylene glycol), chitosan, and polyvinylpyrrolidone, were modelled by searching for their 2D structures on the PubChem website (https://pubchem.ncbi.nlm.nih.gov/; accessed on 18 August 2023) as shown in Figure 4. To determine the lowest energy conformer in polymeric structures such as dimers and trimers, an advanced systematic rotor search was performed using Avogadro 1.2. software [43]. This study allowed us to explore the conformational space of polymeric structures and identify the arrangements of low-energy atoms. Once the global minimal structures were identified, they were saved in .gjf format and further optimized using density functional theory (DFT). Because Standard DFT methods often struggle to accurately describe dispersion interactions, which are important in systems like hydrogels, in this study the exchange-correlation functional ωB97X-D, which includes both large and short dispersion energy corrections [30], and the base ensemble 6-311G(2d,p) in Gaussian view 6 were used. 

The use of DFT in this study provides a theoretical framework for designing hydrogels with optimal CO_2_ absorption capacity, paving the way for further research and development in this field. It is important to note that the specific details and methodologies of the study may vary depending on the research context and objectives [28].

### 4.2. Computational Analysis of Polymers

The structural stability of the systems was ensured by conducting vibrational frequency calculations to confirm the absence of imaginary frequencies within all compounds. Following this validation, optimization of these structures was performed at the ωB97XD/6-311++G(2d,2p) level of theory to facilitate the formation of complexes with carbon dioxide (CO_2_) at the same theoretical level. The determination of the minimum energy complex involved molecular dynamics calculations, employing the Autodock Vina tool [43]. To calculate the total binding energies of the optimized polymers, the following equation was used:

(1) $$∆E_{T}=E^{\left(A,B\right)}-\left(E^{A}-E^{B}\right)+BSSE$$

where $E^{A}\;and\;E^{B}$ represent the electronic energy of molecules A and B, respectively; $E^{\left(A,B\right)}$ represents the electronic energy of the A, B complex, and BSSE represents the overlap error of the base set. A negative value for the change in total energy (∆ET) indicates a favorable interaction between two molecules and an exothermic process [44,45]. Similarly, the thermodynamics of complexations was studied using the Gibbs equation as follows:

(2) $$∆G_{binding}=∆H_{binding}-T∆S_{binding}$$

(3) $$∆H_{binding}=H_{complex}-\left(H_{monom}+H_{dimer}\right)$$

(4) $$∆S_{binding}=S_{complex}-\left(S_{monom}+S_{dimer}\right)$$

In this context, H and S represent the enthalpy and entropy values of the complex, drug, and dimer, respectively. These parameters are calculated at a standard temperature of 298.15 K and a pressure of 1 atm, with data extracted from the results of frequency calculations. A negative ΔE_T_ value signifies a favorable interaction between two molecules, indicative of an exothermic process. Likewise, H and S pertain to the enthalpy and entropy values of the complex, CO_2_, and the hydrogel, all computed under the same standard conditions. A negative ΔG value denotes the spontaneous formation of the complex at the specified temperature, ensuring the spontaneous absorption of CO_2_ within the hydrogel. A negative ΔH value indicates an exothermic process, while positive entropy values suggest an increase in degrees of freedom, signifying that the inclusion complex involves low-energy molecular interactions.

This comprehensive study encompasses a range of temperatures, enabling the construction of a free energy profile that will pinpoint the optimal temperature gradient for absorption and desorption processes. Additionally, the stability of these complexes in solution was assessed through simulation utilizing the CPCM (Conductor Polarizable Continuum Model) solvation method, ensuring a thorough examination of their behavior in a liquid environment [37].

## Figures and Tables

**Table 1:** Binding energy, free energy, enthalpy, and interaction entropy for studied complexes at ωB97X-D/6-311G(2d,p).

Complex	ΔΔG (kcal/mol)	ΔΔH (kcal/mol)	ΔΔS (Cal/mol)	ΔEb (kcal/mol)	Predicted Absorption Yes/No	Experimental Work
Chitosan–CO_2_	−3.50	−11.21	−25.88	−5.41	Yes	[24]
PVP–CO_2_	−3.12	−9.99	−23.07	−4.83	Yes	[25]
EG–CO_2_	−1.98	−6.34	−14.64	−3.06	Yes	[21]
HEAC–CO_2_	−2.78	−8.91	−20.56	−4.29	Yes	No

**Table 2:** Temperature dependence of Gibbs energy at 1 atm, calculated at ωB97X-D/6-311G(2d,p).

Complex/T (°C)	25 °C	100 °C	200 °C	Transition T °C
Chitosan–CO_2_	−3.5	−1.55	1.04	160.00
PVP–CO_2_	−3.12	−1.38	0.93	159.88
PEG–CO_2_	−1.98	−0.88	0.59	159.91
HEAC–CO_2_	−2.78	−1.24	0.82	160.22

## Data Availability

The data presented in this study are openly available in article.
